# Mortality, Causes of Death and Associated Factors Relate to a Large HIV Population-Based Cohort

**DOI:** 10.1371/journal.pone.0145701

**Published:** 2015-12-30

**Authors:** César Garriga, Patricia García de Olalla, Josep M. Miró, Inma Ocaña, Hernando Knobel, Maria Jesús Barberá, Victoria Humet, Pere Domingo, Josep M. Gatell, Esteve Ribera, Mercè Gurguí, Andrés Marco, Joan A. Caylà, on behalf of Barcelona HIV/AIDS working group

**Affiliations:** 1 Epidemiology Service, Public Health Agency of Barcelona, Barcelona, Spain; 2 Spanish Field Epidemiology Training Programme, National Centre for Epidemiology, Instituto de Salud Carlos III, Madrid, Spain; 3 CIBER Epidemiología y Salud Pública (CIBERESP), Madrid, Spain; 4 Hospital Clinic- August Pi i Sunyer Biomedical Research Institute, University of Barcelona, Barcelona, Spain; 5 Infectious Diseases, Hospital Vall de Hebron, Universitat Autònoma de Barcelona, Barcelona, Spain; 6 Internal Medicine-Infectious Diseases, Hospital del Mar, Barcelona, Spain; 7 Direcció General de Serveis Penitenciaris i de Rehabilitació, Departament de Justícia, Barcelona, Spain; 8 Infectious Diseases Unit, Hospital de la Santa Creu i Sant Pau, Universitat Autònoma de Barcelona, Barcelona, Spain; University of New South Wales, AUSTRALIA

## Abstract

**Introduction:**

Antiretroviral therapy has led to a decrease in HIV-related mortality and to the emergence of non-AIDS defining diseases as competing causes of death. This study estimates the HIV mortality rate and their risk factors with regard to different causes in a large city from January 2001 to June 2013.

**Materials and Methods:**

We followed-up 3137 newly diagnosed HIV non-AIDS cases. Causes of death were classified as HIV-related, non-HIV-related and external. We examined the effect of risk factors on survival using mortality rates, Kaplan-Meier plots and Cox models. Finally, we estimated survival for each main cause of death groups through Fine and Gray models.

**Mortality Results:**

182 deaths were found [14.0/1000 person-years of follow-up (py); 95% confidence interval (CI):12.0–16.1/1000 py], 81.3% of them had a known cause of death. Mortality rate by HIV-related causes and non-HIV-related causes was the same (4.9/1000 py; CI:3.7–6.1/1000 py), external was lower [1.7/1000 py; (1.0–2.4/1000 py)].

**Survival Results:**

Kaplan-Meier estimate showed worse survival in intravenous drug user (IDU) and heterosexuals than in men having sex with men (MSM). Factors associated with HIV-related causes of death include: IDU male (subHazard Ratio (sHR):3.2; CI:1.5–7.0) and <200 CD4 at diagnosis (sHR:2.7; CI:1.3–5.7) versus ≥500 CD4. Factors associated with non-HIV-related causes of death include: ageing (sHR:1.5; CI:1.4–1.7) and heterosexual female (sHR:2.8; CI:1.1–7.3) versus MSM. Factors associated with external causes of death were IDU male (sHR:28.7; CI:6.7–123.2) and heterosexual male (sHR:11.8; CI:2.5–56.4) versus MSM.

**Conclusion and Recommendation:**

There are important differences in survival among transmission groups. Improved treatment is especially necessary in IDUs and heterosexual males.

## Introduction

The survival of HIV-infected people underwent a remarkable improvement after the introduction of highly active antiretroviral therapy (HAART) in 1996 [1–3]. Since then, the natural history of HIV disease as well as the morbidity and the mortality patterns have changed. HIV-infected people are now experiencing immune reconstitution and prolonged survival as a result of HAART. In parallel, HAART has undergone a great evolution: the new antiretrovirals with milder side-effects have facilitated adherence to treatment and have contributed to an increased life expectancy [4]. Clinical trials and observational studies demonstrate that the introduction of HAART and its continuous implementation have redefined the HIV epidemic [5–8].

The changes in HIV transmission routes have also contributed to epidemic evolution. In the 80s and 90s, the HIV and the heroin epidemics were closely related to one another in Western Europe [9]. Intravenous drug users (IDU) and their partners constituted the greatest number of new infections. The causes of death among IDUs were frequently related to infections, drug dependence, violence and suicide, along with a poor adherence to the medical treatments [8, 10–13].

HIV infection has more recently acquired the features of a sexually transmitted epidemic, where the group with the highest number of infections is men who have sex with men (MSM) [14, 15]. Over previous decades, the higher survival rate of HIV-patients along with the changes in the epidemic transmission route led to the onset of non-HIV-related causes of death, among them: cancer, liver and cardiovascular diseases [1, 16, 17]. The identification and better understanding of these new endpoints may facilitate the development of new clinical guidelines and practices for the management of HIV-infected patients.

Here, we aim to inform the development of new prevention plans by studying a large cohort of HIV-non-AIDS population. Our specific objectives are: 1) to calculate mortality rates according to the causes of death and their association to transmission categories and socio-demographic factors, 2) to determine factors related with worse survival in the defined population-based cohort and 3) to identify factors associated with worse survival in three groups, formed on the basis of cause of death (HIV-related, non-HIV-related and external).

## Materials and Methods

### Study design, setting and participants

We undertook an observational study in a population-based cohort of HIV-infected patients residing in Barcelona aged 18 years and older. The study spanned the period between 2001 and 30^th^ of June 2013 (the end of follow up). Patients first diagnosed with HIV at the AIDS stage (less than 200 CD4 cells/μl and become ill with an opportunistic infection) were not included in the study [18]. The aim was to assess the impact of the risk factors on HIV-related or a non-HIV-related outcome before AIDS was present.

The cases were reported to the surveillance system of the Public Health Agency of Barcelona between 2001 and 2012. Reporting of new cases of HIV infection was voluntary between 2001 and 2009, but became mandatory for physicians from 2010. From then on the Barcelona HIV surveillance system became an active system that collects data provided by doctors, hospital discharges, the sexually transmitted infections registry, and mortality databases regarding patients diagnosed with HIV/AIDS.

### Data collection

Clinicians completed a standard data collection form and used nominal identification. Socio-demographic factors considered in the study were: sex, date and country of origin, completed educational level and district household income at diagnosis [index calculated from (1) educational level of the population, (2) work status, (3) the amount of cars, (4) the power of newly bought cars, and (5) second-hand housing prices; it is measured as units standard: low-medium (<100), high (100–159) and very high (>159)] [19], HIV transmission category (IDU, MSM and heterosexual) and clinical data [CD4 cell count/μl; viral load (RNA copies/mL), hepatitis B surface antigen (HBsAg) and anti-HCV antibodies, year of HIV diagnosis]. A compound variable was constructed combining sex and HIV transmission. This variable takes into account the confounding of effects between sex and HIV transmission categories, resulting from the fact that the MSM transmission category only includes men, and that there are more IDU males than females [20].

Date of death was collected from the municipal registry of Barcelona city between January 2001 and June 2013. The administrative censoring date was 30^th^ June 2013. Patients with a listing or delisting date in the municipal registry after this date were classified as alive at the end of the study. Subjects were considered lost to follow-up if they did not have a listing or delisting date after the administrative censoring date. Individuals were censored at the midpoint between their last recorded date and the administrative censoring date. Subjects with no registered date of death in the Catalonia region were classified as alive or loss to follow-up. Information on the status of the subjects lost to follow-up due to relocation was unavailable.

Specific cause of death was searched for in the Barcelona city death registry. The causes of death were coded using the International Classification of Diseases 10th revision (ICD-10) [21]. Specific causes of death were classified in three main groups: HIV-related (B20-B24, B44.9, C83.7, C85.9, and tuberculosis codes A15-A19), non-HIV-related (remaining codes not included in HIV-related or external groups) and external (overdose, suicide and accident codes: F19.2, Y19.9 and, X00-X99).

### Statistical analysis

#### Descriptive analysis

Socio-demographic and clinical frequencies were calculated. Median and interquartile ranges (IQR) for continuous variables were obtained (i.e., age, CD4 counts and viral load).

#### Mortality

Incidence density and mortality rates were calculated as person-years of follow-up (py) per thousand with 95% confidence interval (CI). Mortality rates were obtained for specific and grouped causes of death according to sex, country of origin, educational level, district household income, transmission category (by sex), CD4 counts, and year of HIV diagnosis.

Survival time was estimated as the number of years between the date of HIV diagnosis and the date of death or the end of follow-up. Median and IQR survival times were obtained from HIV-related, non-HIV-related, and external causes of death. These values were compared using the Kruskal-Wallis non-parametric test to check if there were different survival times according to the causes of death.

#### Survival analysis

Kaplan-Meier survival analysis was conducted to estimate and to graph survival for various factors (socio-demographic and clinical). Next, survival curves were compared using the log-rank test with the null hypothesis of a common survival curve (*P* values < 0.05 indicating a statistically significant difference between categories of the variables analysed). Multivariable analysis was carried out using Cox proportional hazards regression model after adjustment for confounding factors (age, country of origin, educational level, HIV transmission category by sex and CD4 counts at HIV diagnosis). The effect of the factors on survival was assessed by hazard ratios (HR) and their CI. Statistically significant factors in the univariable model were included in the multivariable model.

Three multivariable Fine and Gray regressions were fitted to estimate the adjusted effect of factors on the different outcomes (HIV-related, non-HIV-related and external) accounting for competing risks among all groups (the three outcome groups and the group with unidentified cause of death) [22]. These analyses were adjusted for any confounders. Sub-distributional hazard ratios were obtained. Unlike the hazard ratio, sub-distributional hazard represent the ordering and not a numerical value for the ratio [23]. A sensitivity Fine and Gray regression was performed to estimate the effect of the factors associated with unavailable cause of death, following diagnosis for HIV. In addition, a sensitivity analysis was performed to evaluate immortal time bias including only those patients with administrative censoring together with who died after one-year follow-up (n = 266). Analyses were performed using SPSS v.22.0 for descriptive analysis and Stata v.13 for Cox, Fine and Gray regression models.

### Ethical considerations

HIV is a mandatory notifiable infection for health professionals in compliance with Article 13 of the law 67/2010 (25th May 2010) of the Health Department of Generalitat de Catalunya. All data were treated in a strictly confidential manner according to the ethical principles of the Helsinki Declaration of 1964 revised by the World Medical Organization in Fortaleza, 2013 [24], and Law 15/1999 of Personal Data Protection in Spain [25]. Patient information was anonymized and de-identified prior to analysis.

## Results

### Study population

We followed-up 3137 new HIV non-AIDS diagnoses which represented 12960 py. 85.9% of the cohort was male (Table 1). 61.0% of the patients were MSM. At diagnosis, the median age was 33 years (IQR 28–40 years), the median CD4 count was 399 cells/μL (IQR 245–579 cells/μL) and the median viral load was 45500 RNA copies/mL (IQR 11500–148300 RNA copies/mL).

**Table 1:** Socio-demographic and clinical features of HIV-non-AIDS patients. Barcelona city, 2001–2012.

		HIV-infected individuals	
		n	
**Socio-demographic features**			
	**Sex (%)**		
	Male	**2694**	(85.9)
	Female	**443**	(14.1)
	**Median age (IQR)**	**3137**	33 (28–40)
	**Country of origin (%)**		
	Spaniards	**1732**	(55.2)
	Immigrants	**1403**	(44.7)
	Unknown	**2**	(0.1)
	**Education level completed (%)**		
	Illiteracy/Primary	**573**	(18.3)
	Lower secondary	**689**	(22.0)
	Upper secondary	**887**	(28.3)
	University	**911**	(29.0)
	Unknown	**77**	(2.5)
	**District household income** ^**a**^ **(%)**		
	Low-medium	**1755**	(55.9)
	High	**1111**	(35.4)
	Very high	**226**	(7.2)
	Unknown	**45**	(1.4)
	**HIV transmission category (%)**		
	IDU Male	**253**	(8.1)
	IDU Female	**75**	(2.4)
	MSM non-IDU	**1913**	(61.0)
	Heterosexual Male	**378**	(12.0)
	Heterosexual Female	**347**	(11.1)
	Unknown	**171**	(5.5)
**Clinical features** ^**b**^			
	**CD4 (cells/μl)** ^**c**^ **(%)**		
	<200	**478**	(15.2)
	200–349	**581**	(18.5)
	350–499	**621**	(19.8)
	≥500	**899**	(28.7)
	Unknown	**558**	(17.8)
	**Viral load (RNA copies/mL)** ^**d**^ **(%)**		
	<50	**69**	(2.2)
	50–499	**103**	(3.3)
	500–29999	**898**	(28.6)
	≥30000	**1408**	(44.9)
	Unknown	**659**	(21.0)
	**HbsAg (%)**		
	Positive	**179**	(5.7)
	Negative	**2183**	(69.6)
	Unknown	**775**	(24.7)
	**Anti-HCV antibodies (%)**		
	Positive	**351**	(11.2)
	Negative	**2024**	(64.5)
	Unknown	**762**	(24.3)
	**Year of HIV diagnosis**		
	2001	168	(5.4)
	2002	221	(7.0)
	2003	211	(6.7)
	2004	247	(7.9)
	2005	234	(7.5)
	2006	274	(8.7)
	2007	251	(8.0)
	2008	280	(8.9)
	2009	257	(8.2)
	2010	340	(10.8)
	2011	333	(10.6)
	2012	321	(10.2)
**Total**		**3137**	

IQR, interquartile range; MSM, men who have sex with other men; IDU, injecting drug user; HBsAg, hepatitis B surface antigen and HCV, hepatitis C virus.

^a^ 2013 Barcelona household income according to district where the person diagnosed with HIV lived.

^b^ At the time of HIV diagnosis.

^c^ <200 CD4 counts highlights severe immunodeficiency.

^d^ <50 RNA copies/mL highlights suppressive control of HIV.

#### Educational level

There was a significantly higher proportion of IDUs in the group of patients with lower educational level (26.0%) than in the higher education level (1.5%). In addition, patients in the lower educational level group had lower CD4 counts at diagnosis (<200 CD4 cells/μl) than patients in the higher educational level group (19.9% vs. 12.0%). The proportion of immigrants having low educational level (14.5%) was lower than in Spanish-born (21.4%).

### Mortality rate and causes of death

182 deaths were identified (14.0/1000 py; CI: 12.0―16.1/1000 py), 148 (81.3%) of them had a known cause of death, and 34 had an unknown cause of death. Educational level was the only statistically significant difference between known cause of death group and unknown cause of death group (*P*<0.01).

There was not a statistical difference in mortality rates among cause of death groups (*P* = 0.5). The mortality rate by HIV-related causes and non-HIV-related causes was the same (4.9/1000 py; CI: 3.7–6.1/1000 py) (Table 2). Individuals who died from external causes presented a lower mortality rate (1.7/1000 py; CI: 1.0–2.4/1000 py). We did not detect any external cause of death among women. In addition, Table 2 also shows crude mortality rates weighted per category in main covariates.

**Table 2:** Mortality rates. Barcelona city, January 2001- June 2013.

Causes of death			All causes		HIV-related		Non-HIV-related		External	
	n	py	deaths	WA (95% CI)	deaths	WA (95% CI)	deaths	WA (95% CI)	deaths	WA (95% CI)
**Total**	3137	12960	182	14.0 (12.0–16.1)	63	4.9 (3.7–6.1)	63	4.9 (3.7–6.1)	22	1.7 (1.0–2.4)
**Socio-demographic features**										
**Sex**										
Male	2694	10769	144	11.5 (9.6–13.3)	52	4.1 (3.0–5.3)	45	3.6 (2.5–4.6)	22	1.8 (1.0–2.5)
Female	443	2190	38	2.5 (1.7–3.2)	11	0.7 (0.3–1.1)	18	1.2 (0.6–1.7)	0	─
**Age**										
18–29 years	979	4277	21	1.5 (0.9–2.2)	10	0.7 (0.3–1.2)	3	0.2 (0.0–0.5)	4	0.3 (0.0–0.6)
30–39 years	1311	5572	60	4.5 (3.4–5.6)	23	1.7 (1.0–2.4)	13	1.0 (0.4–1.5)	11	0.8 (0.3–1.3)
≥40 years	847	3111	101	8.8 (7.1–10.4)	30	2.6 (1.7–3.5)	47	4.1 (2.9–5.2)	7	0.6 (0.2–1.1)
**Country of origin**										
Spaniards	1732	7230	151	11.5 (9.7–13.4)	48	3.7 (2.6–4.7)	57	4.4 (3.2–5.5)	20	1.5 (0.9–2.2)
Immigrants	1403	5719	31	2.4 (1.6–3.3)	15	1.2 (0.6–1.8)	6	0.5 (0.1–0.8)	2	0.2 (0.0–0.4)
Unknown	2	12	0	─	0	─	0	─	0	─
**Education level completed**										
Illiteracy/Primary	573	2652	78	5.4 (4.2–6.5)	30	2.1 (1.3–2.8)	30	2.1 (1.3–2.8)	8	0.6 (0.2–0.9)
Lower secondary	689	2946	36	2.7 (1.8–3.6)	14	1.0 (0.5–1.6)	11	0.8 (0.3–1.3)	10	0.7 (0.3–1.2)
Upper secondary	887	3597	34	2.7 (1.8–3.6)	9	0.7 (0.2–1.2)	12	0.9 (0.4–1.5)	4	0.3 (0.0–0.6)
University	911	3488	17	1.4 (0.7–2.1)	7	0.6 (0.2–1.0)	7	0.6 (0.2–1.0)	0	─
Unknown	77	277	17	1.5 (0.8–2.2)	3	0.3 (0.0–0.6)	3	0.3 (0.0–0.6)	0	─
**District household income** ^**a**^										
Low-medium	1755	7351	113	8.6 (7.0–10.2)	41	3.1 (2.2–4.1)	41	3.1 (2.2–4.1)	12	0.9 (0.4–1.4)
High	1111	4524	47	3.7 (2.6–4.7)	16	1.3 (0.6–1.9)	15	1.2 (0.6–1.8)	9	0.7 (0.2–1.2)
Very high	226	933	14	1.1 (0.5–1.6)	5	0.4 (0.0–0.7)	6	0.5 (0.1–0.8)	1	0.1 (0.0–0.2)
Unknown	45	152	8	0.8 (0.2–1.3)	1	0.1 (0.0–0.3)	1	0.1 (0.0–0.3)	0	─
**HIV transmission category**										
IDU Male	253	1278	50	3.2 (2.3–4.0)	18	1.1 (0.6–1.7)	13	0.8 (0.4–1.3)	13	0.8 (0.4–1.3)
IDU Female	75	396	14	0.8 (0.4–1.3)	3	0.2 (0.0–0.4)	7	0.4 (0.1–0.7)	0	─
MSM non-IDU	1913	7371	38	3.1 (2.1–4.1)	15	1.2 (0.6–1.9)	12	1.0 (0.4–1.6)	2	0.2 (0.0–0.4)
Heterosexual Male	378	1508	43	3.4 (2.4–4.4)	14	1.1 (0.5–1.7)	16	1.3 (0.7–1.9)	7	0.6 (0.1–1.0)
Heterosexual Female	347	1697	23	1.5 (0.9–2.1)	8	0.5 (0.2–0.9)	10	0.7 (0.2–1.1)	0	─
Unknown	171	709	14	1.1 (0.5–1.6)	5	0.4 (0.0–0.7)	5	0.4 (0.0–0.7)	0	─
**Clinical features** ^**b**^										
**CD4 (cells/μl)** ^**c**^										
<200	478	1971	58	4.5 (3.3–5.6)	28	2.2 (1.4–3.0)	18	1.4 (0.8–2.0)	4	0.3 (0.0–0.6)
200–349	581	2401	28	2.2 (1.4–3.0)	7	0.5 (0.1–0.9)	10	0.8 (0.3–1.2)	5	0.4 (0.0–0.7)
350–499	621	2459	18	1.4 (0.8–2.1)	7	0.6 (0.1–1.0)	6	0.5 (0.1–0.9)	2	0.2 (0.0–0.4)
≥500	899	3627	32	2.5 (1.7–3.4)	11	0.9 (0.4–1.4)	9	0.7 (0.2–1.2)	5	0.4 (0.0–0.7)
Unknown	558	2501	46	3.3 (2.3–4.2)	10	0.7 (0.3–1.2)	20	1.4 (0.8–2.0)	6	0.4 (0.1–0.8)
**Year of HIV diagnosis**										
2001	168	1039	23	1.2 (0.7–1.7)	7	0.4 (0.1–0.6)	6	0.3 (0.1–0.6)	2	0.1 (0.0–0.2)
2002	221	1745	37	1.5 (1.0–2.0)	14	0.6 (0.3–0.9)	11	0.4 (0.2–0.7)	5	0.2 (0.0–0.4)
2003	211	1519	23	1.0 (0.6–1.4)	9	0.4 (0.1–0.7)	11	0.5 (0.2–0.8)	2	0.1 (0.0–0.2)
2004	247	1568	26	1.3 (0.8–1.8)	8	0.4 (0.1–0.7)	11	0.6 (0.2–0.9)	4	0.2 (0.0–0.4)
2005	234	1375	14	0.8 (0.4–1.2)	3	0.2 (0.0–0.3)	5	0.3 (0.0–0.5)	3	0.2 (0.0–0.3)
2006	274	1452	19	1.1 (0.6–1.7)	4	0.2 (0.0–0.5)	9	0.5 (0.2–0.9)	1	0.1 (0.0–0.2)
2007	251	1115	9	0.6 (0.2–1.1)	4	0.3 (0.0–0.6)	1	0.1 (0.0–0.2)	1	0.1 (0.0–0.2)
2008	280	1027	9	0.8 (0.3–1.3)	5	0.4 (0.1–0.8)	4	0.3 (0.0–0.7)	0	─
2009	257	748	6	0.7 (0.1–1.2)	4	0.4 (0.0–0.9)	2	0.2 (0.0–0.5)	0	─
2010	340	731	8	1.2 (0.4–2.0)	4	0.6 (0.0–1.2)	0	─	2	0.3 (0.0–0.7)
2011	333	445	7	1.7 (0.4–2.9)	1	0.2 (0.0–0.7)	3	0.7 (0.0–1.5)	1	0.2 (0.0–0.7)
2012	321	194	1	0.5 (0.0–1.6)	0	─	0	─	1	0.5 (0.0–1.6)

py, person-years follow-up in a category; WA, Weighted average of crude mortality rate in categories; CI, confidence intervals; MSM, men who have sex with other men; IDU, injecting drug user; HBsAg, hepatitis B surface antigen and HCV, hepatitis C virus.

WA = (deaths/py) x (n/3137); deaths/py = mortality rate

^a^ 2013 Barcelona household income according to district where the person diagnosed of HIV lived

^b^ At the time of HIV diagnosis

^c^ <200 CD4 counts highlights severe immunodeficiency

We compared mortality among Spanish-born and immigrants (11.5/1000 py; CI: 9.7–13.4/1000 py vs. 2.4/1000 py; 1.6 py– 3.3/1000 py, respectively). We observed a lower mortality rate for each cause of death in the group of immigrants, when compared to the group of Spanish-born people (Table 2). Moreover, we found that immigrants did not suffer from non-HIV-related causes of death such as other infections, cancer or liver disease (data not shown). The proportion of people, older than 49 years of age, living with HIV, was three times higher in the Spanish-born group than in the immigrant group (12.1% vs. 4.3%, respectively).

In addition, we analysed CD4 counts at HIV diagnosis. We found that mortality rates were higher in patients with CD4 count ≤ 200 cells/μL for main causes of death (Table 2). Furthermore, we assessed the influence of educational level on mortality rates. We detected higher mortality rates in individuals with lower educational level (Table 2). Finally, we did not find differences in crude mortality rates for the year of HIV diagnosis among years with voluntary reporting (2001–2009) and compulsory (2010–2012) (Table 2).

### Survival outcomes

**Fig 1:**
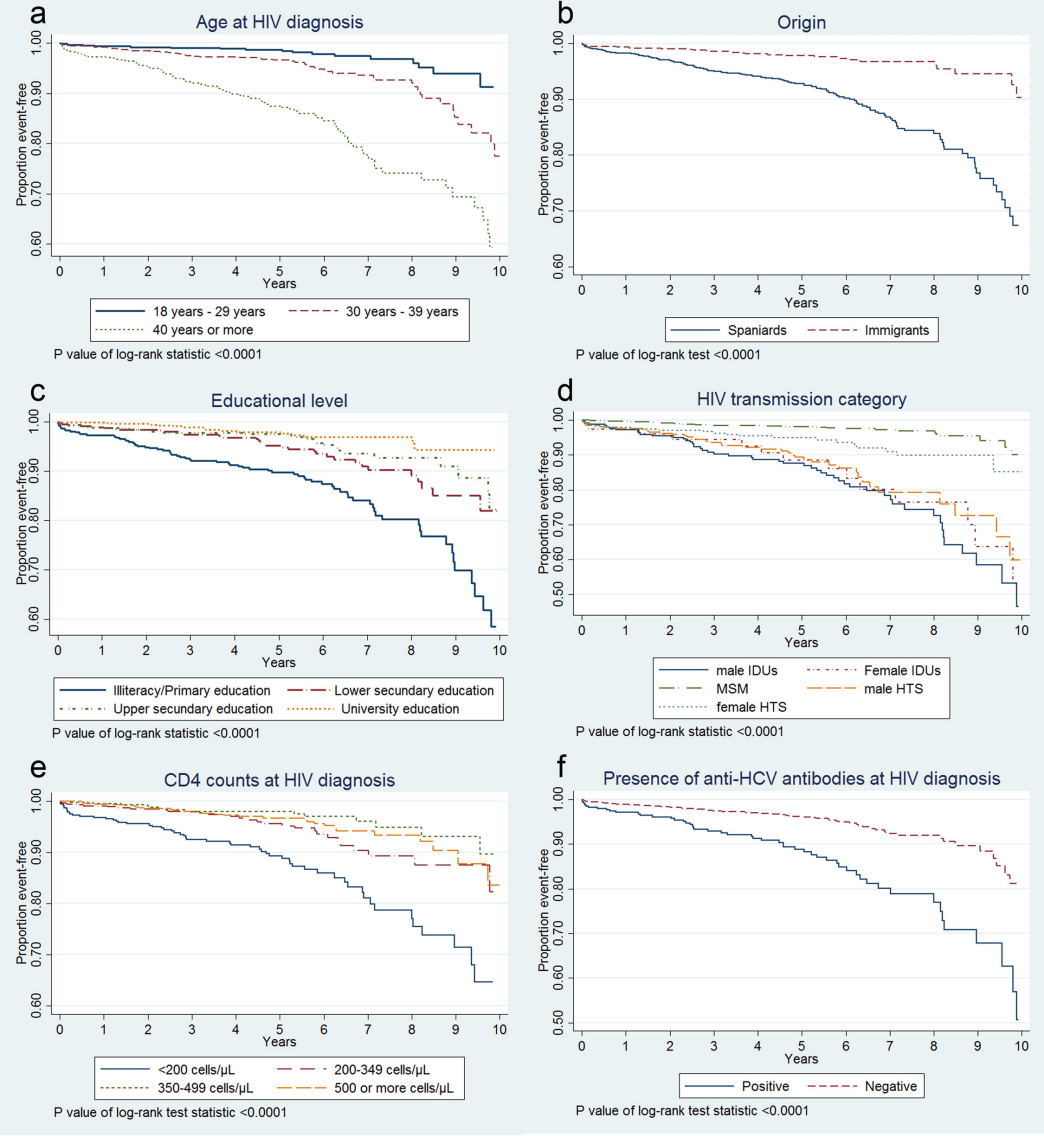
Survival curves according to age, origin, educational level, transmission category, CD4, and anti-HCV antibodies. Survival probabilities according to: (a) age at HIV diagnosis, (b) country of origin, (c) educational level, (d) HIV transmission category and sex (IDUs: injecting drug users, MSM: men who have sex with men, and HTS: heterosexuals), (e) CD4 cell count/μl at HIV diagnosis and (f) anti-HCV antibodies at HIV diagnosis.

The cumulative survival probability 12 years after diagnosis was 83.1% (CI 78.1%-87.1%). We found significant differences (*P* < 0.01) in the Kaplan-Meier survival analysis by age at HIV diagnosis, country of origin, educational level, district household income, HIV transmission category (by sex), CD4 and anti-HCV antibodies (Fig 1). Furthermore, we observed a strong inverse relationship between age at HIV diagnosis and CD4 counts (*P* linear association = 5.4 x 10^−21^), i.e. higher age was related to lower CD4 level.

### Factors associated with survival

Table 3 show the factors associated with worse survival. Spanish-born patients showed higher mortality than immigrants. We also observed that those with illiteracy or primary education only had worse survival than those having university education, in turn we did not find differences according to district household income among HIV-infected residents at the time of diagnosis.

**Table 3:** Cox proportional hazards model showing factors associated with survival in a cohort of 3137 HIV-non-AIDS patients. Barcelona city, January 2001 –June 2013.

	Univariable analysis			Multivariable analysis^b^		
Independent variables^a^	Hazard ratio (95% CI)		*P* value (Wald test)	Hazard ratio (95% CI)		*P* value (Wald test)
**Age at HIV diagnosis (continuous variable 5-year groups)** ^**b**^	1.4	(1.3–1.5)	<0.001	1.3	(1.2–1.4)	<0.001
**Country of origin (Immigrants)** ^**b**^						
Spaniards	3.9	(2.7–5.8)	<0.001	2.3	(1.5–3.5)	<0.001
**Educational level completed (University education)** ^**b**^						
Illiteracy/Primary education	6.1	(3.6–10.3)	<0.001	2.2	(1.2–3.9)	0.007
Lower secondary education	2.5	(1.4–4.5)	0.002	1.5	(0.8–2.8)	0.174
Upper secondary education	2.0	(1.1–3.5)	0.023	1.6	(0.9–2.8)	0.143
Unknown	15.1	(7.7–29.7)	<0.001	7.6	(3.8–15.5)	<0.001
**District household income** ^**c**^ **(Very high)** ^**b**^						
Low-medium	1.0	(0.6–1.7)	0.959			
High	0.7	(0.4–1.2)	0.214			
Unknown	3.9	(1.6–9.3)	0.002			
**HIV transmission category (MSM non-IDU)** ^**b**^						
IDU Male	7.6	(5.0–11.7)	<0.001	4.1	(2.6–6.5)	<0.001
IDU Female	6.2	(3.3–11.4)	<0.001	3.3	(1.7–6.3)	<0.001
Heterosexual Male	5.8	(3.7–9.0)	<0.001	2.6	(1.6–4.1)	<0.001
Heterosexual Female	2.6	(1.5–4.3)	<0.001	1.7	(1.0–2.9)	0.064
Unknown	3.8	(2.0–6.9)	<0.001	2.3	(1.2–4.3)	0.012
**CD4 at diagnosis (≥500)**						
<200^d^	3.2	(2.1–5.0)	<0.001	1.7	(1.1–2.7)	0.016
200–349	1.3	(0.8–2.1)	0.336	1.1	(0.6–1.8)	0.790
350–499	0.8	(0.4–1.4)	0.416	0.7	(0.4–1.3)	0.263
Unknown	2.0	(1.3–3.1)	0.003	1.4	(0.9–2.2)	0.183
**Viral load at diagnosis (<50)** ^**b**^ ^,^ ^**e**^						
50–499	1.4	(0.3–7.7)	0.687			
500–29 999	2.0	(0.5–8.4)	0.328			
≥30 000	1.9	(0.5–7.8)	0.370			
Unknown	3.2	(0.8–13.3)	0.101			
**Anti-HCV (Negative)** ^**b**^						
Positive	3.0	(2.1–4.3)	<0.001			
Unknown	1.2	(0.8–1.7)	0.349			
**Year of HIV diagnosis (2007)**						
2001	2.2	(1.0–4.8)	0.049			
2002	1.4	(0.7–3.1)	0.352			
2003	1.3	(0.6–2.9)	0.513			
2004	1.7	(0.8–3.6)	0.186			
2005	1.1	(0.5–2.6)	0.790			
2006	1.5	(0.7–3.4)	0.305			
2008	1.1	(0.4–2.8)	0.843			
2009	1.0	(0.3–2.8)	0.973			
2010	1.3	(0.5–3.4)	0.615			
2011	1.7	(0.6–4.8)	0.292			
2012	0.4	(0.0–3.1)	0.377			

CI, confidence intervals; MSM, men who have sex with other men; IDU, injecting drug user; and HCV, hepatitis C virus.

^a^ Reference categories are shown in parentheses.

^b^ *P* values (Pseudo-Likelihood Ratio test) <0.05 by factors in crude analysis and as a whole in multivariable analysis.

^c^ 2013 Barcelona household income according to district where the person diagnosed of HIV lived.

^d^ <200 CD4 counts highlights severe immunodeficiency.

^e^ <50 RNA copies/mL highlights suppressive control of HIV.

We evaluated immortal time bias using a Cox proportional hazards model, with patients with administrative censoring and with those who died after one-year of follow-up (n = 266). We found a statistically significant association with ageing (HR: 1.1; 95% CI: 1.0–1.2); and IDU male and heterosexual male vs. MSM non-IDU (HR: 1.9; 95% CI: 1.0–3.6 and HR: 2.1; 95% CI: 1.1–3.8, respectively).

We observed that 1092 of 3137 had incomplete data for some of the variables included in the adjusted model (age, country of origin, educational level, district household income, HIV transmission category, CD4 counts, and antibodies anti-HCV). We validated the hazard ratios direction and value with a univariable model excluding the missing values for the adjustment variables. Equal statistically significant hazard ratio was obtained in this comparison for age, educational level, district household income, CD4+ counts, and positive to antibodies anti-HCV. Similar significant hazard ratios were calculated comparing Spanish-born vs. immigrants (HR: 3.5; 95% CI: 2.4–5.1); and HIV transmission category, e.g.: HTS female vs. MSM non-IDU (HR: 2.6; 95% CI: 1.5–4.3). This suggests that missing values in these variables are not modifying the direction of hazard ratios in the models that did not exclude missing values.

### Prognostic factors

The factors associated with HIV-related causes of death were: older age at HIV diagnosis, 20% increase in death risk corresponding to each 5-year period; low educational level; IDU male and CD4<200 (Table 4). In the unadjusted analysis we observed a higher risk of death by HIV-related causes of death in those with illiteracy or primary education than those with university studies. In addition we observed a non-statistically significant reduction in risk from lower to higher schooling.

**Table 4:** Factors associated with HIV-related causes of death, non-HIV-related and external following diagnosis for HIV. Competing risk hazards for univariable and multivariable models. Barcelona city, January 2001 –June 2013.

	HIV-related (63 deaths)				non-HIV-related (63 deaths)				external (22 deaths)			
	Crude analysis		Adjusted analysis		Crude analysis		Adjusted analysis		Crude analysis		Adjusted analysis	
Independent variables^a^ ^,^ ^b^	sHR	95% CI	sHR	95% CI	sHR	95% CI	sHR	95% CI	sHR	95% CI	sHR	95% CI
**Age at HIV diagnosis (continuous variable)**	**1.3** ^**c**^	**(1.2–1.4)**	**1.2**	**(1.1–1.3)**	**1.5**	**(1.4–1.6)**	**1.5**	**(1.4–1.7)**	1.1	(0.9–1.3)	1.0	(0.8–1.2)
**Country of origin (Immigrants)**												
Spaniards	**2.5**	**(1.4–4.4)**	1.4	(0.7–2.6)	**7.4**	**(3.2–17.0)**	**3.8**	**(1.6–8.8)**	**7.7**	**(1.8–32.8)**	3.6	(0.8–15.1)
**Educational level completed (University)**												
Illiteracy/Primary education	**5.3**	**(2.3–12.2)**	**2.7**	**(1.1–6.6)**	**5.1**	**(2.2–11.6)**	1.3	(0.6–3.2)	2.5	(0.7–8.3)	1.0	(0.3–3.5)
Lower secondary education	2.3	(0.9–5.8)	1.6	(0.6–4.1)	1.8	(0.7–4.6)	1.0	(0.4–2.5)	3.0	(1.0–9.5)	1.7	(0.5–5.5)
Upper secondary education	1.2	(0.5–3.4)	1.1	(0.4–2.9)	1.7	(0.7–4.2)	1.2	(0.5–3.0)	1^e^	–	1^e^	–
Unknown	**5.0**	**(1.3–19.7)**	2.8	(0.7–11.4)	**5.1**	**(1.4–18.5)**	1.8	(0.4–8.0)	–	–	–	–
**HIV transmission category (MSM non-IDU)**												
IDU Male	**6.4**	**(3.3–12.6)**	**3.2**	**(1.5–7.0)**	**5.4**	**(2.4–11.9)**	1.7	(0.7–4.3)	**34.3**	**(7.9–149.0)**	**28.7**	**(6.7–123.2)**
IDU Female	3.2	(0.9–10.9)	1.8	(0.5–6.5)	8.8	(3.3–23.3)	2.9	(0.9–9.1)	–	–	–	**–**
Heterosexual Male	**4.4**	**(2.1–9.0)**	1.8	(0.8–4.2)	**6.2**	**(2.9–13.2)**	**2.5**	**(1.1–6.0)**	**16.5**	**(3.4–78.8)**	**11.8**	**(2.5–56.4)**
Heterosexual Female	2.2	(0.9–5.3)	1.2	(0.5–3.0)	**3.3**	**(1.4–7.8)**	**2.8**	**(1.1–7.3)**	–	–	–	–
Unknown	**3.2**	**(1.1–9.0)**	2.4	(0.8–7.1)	4.1	(1.4–11.6)	1.5	(0.5–4.2)	–	–	–	–
**CD4 at diagnosis (≥500)**												
<200^d^	**4.4**	**(2.2–8.8)**	**2.7**	**(1.3–5.7)**	**3.4**	**(1.5–7.7)**	1.6	(0.7–3.9)	1.4	(0.4–5.0)	1.0	(0.2–3.9)
200–349	0.9	(0.4–2.4)	0.8	(0.3–2.2)	1.6	(0.7–4.0)	1.2	(0.5–3.2)	1.5	(0.4–5.2)	1.6	(0.5–5.3)
350–499	0.9	(0.4–2.3)	0.9	(0.4–2.2)	0.9	(0.3–2.6)	0.8	(0.3–2.3)	0.6	(0.1–3.0)	0.8	(0.1–4.6)
Unknown	1.2	(0.5–2.9)	0.8	(0.3–2.0)	**3.0**	**(1.4–6.6)**	**2.3**	**(1.0–5.4)**	1.7	(0.5–5.5)	1.5	(0.5–5.0)
**Anti-HCV (Negative)**												
Positive	**2.1**	**(1.2–3.8)**	–	–	**2.1**	**(1.2–3.8)**	**4.3**	**(1.9–9.8)**	**3.4**	**(1.4–8.6)**	0.4	(0.1–1.4)
Unknown	0.8	(0.4–1.5)	–	–	0.8	(0.4–1.5)	**3.1**	**(1.4–6.5)**	0.7	(0.2–2.3)	0.4	(0.1–1.2)

sHR, subHazard Ratio; CI, confidence intervals; MSM, men who have sex with other men; IDU, injecting drug user; and HCV, hepatitis C virus.

^a^ All the models have been adjusted by age, origin, educational level, HIV transmission, and CD4 counts. A sensitivity analysis was carried out from non-available causes of death. The only associated factor was being older at HIV diagnosis, with each 5 years the death risk increased by 30%.

^b^ Reference categories are shown in parenthesis.

^c^ Bold numbers highlight *P* values<0.05 for Wald test.

^d^ <200 CD4 counts highlights severe immunodeficiency.

^e^ Upper secondary education as reference category.

Factors associated with non-HIV-related causes of death were: older age at HIV diagnosis, 50% increase in death risk corresponding to each 5-year age period; Spanish-born; IDU female (marginally significant) or heterosexual male and female; and presence of anti-HCV antibodies at HIV diagnosis (Table 4).

Factors associated with external causes of death were: IDU male and heterosexual male (Table 4).

Competing risk hazards models with administrative censoring and patients who had died after one-year of follow-up (n = 266) were performed to evaluate potential immortal time bias. The model of HIV-related causes of death was not statistically significant because of the loss in statistical power. The model of non-HIV-related causes of death found a statistically significant association with ageing (sHR: 1.4; 95% CI: 1.2–1.6); heterosexual female vs. MSM non-IDU (sHR: 3.4; 95% CI: 1.1–10.5) and positive vs negative antibodies anti-HCV (sHR: 5.2; 95% CI: 2.0–13.4). The model of external causes of death found a statistically significant association with ageing, and IDU male and heterosexual male vs. MSM non-IDU (sHR: 18.9; 95% CI: 2.7–129.5 and sHR: 12.5; 95% CI: 1.4–110.8, respectively).

## Discussion

### Key results

Because of epidemic and treatment changes in the HAART era we expected to find higher mortality rates for non-HIV-related causes of death group than for HIV-related ones [26, 27]. However, we found equal mortality rates. However, the mortality rates for HIV-related causes of death would have been greater than for non-HIV-related if some of the non-HIV-related causes of death were in fact, either directly or indirectly, a consequence of HIV infection (e.g. cancers could be associated with loss of immunity).

Our unit observed in previous studies that all-cause mortality tends to diminish over the time for every transmission category [28, 29]. Nonetheless, the overall rate (14.0/1000 py) was almost twice that of the general Barcelona city population (range of 7.8–8.3/1000 py for 2009–2013) [30]. We also found higher mortality than the Spanish AIDS research network (10.2/1000 py; CI: 9.1―11.5/1000 py) [17] and slightly greater than a collaborative cohort consisting of by 13 cohorts of Europe and North America of patients starting HAART during 1996–2006 (12.1/1000 py; CI: 11.6―12.7/1000 py) [31]. Most likely, the higher mortality found in this population level study is because we included patients with higher risk of death than those considered in active follow-up cohorts. This is because we include patients not receiving HIV medical care as well as those who are [32].

Differences among transmission groups continue to be important. IDUs are a vulnerable group with respect to death from HIV-related and external causes, although they make up only 10% of new HIV-non-AIDS diagnoses [33]. The IDU males had a higher mortality rate by external causes of death. MSM had higher weighted crude mortality rates for both HIV-related and non-HIV-related causes of death, as well as heterosexual males. In the adjusted model we found higher mortality in heterosexual males and IDUs (male and female) than MSM. These groups have been found to be more likely to be diagnosed at a later stage, related to receiving care for their condition [33, 34].

### Factors associated with survival

#### Age and CD4 counts

Besides lower CD4 counts, the patients with worse survival were also older. Being older at HIV diagnosis was associated with higher mortality by HIV-related and non-HIV-related causes of death. This risk of death was higher for non-HIV-related causes of death suggesting that HIV infection accelerates the progression of other comorbidities. Therefore, classification of some ICD-10 codes as non-HIV-related causes of death might need revision. In addition, other co-morbidities such as hepatitis B or C, and a longer delay in the diagnosis increase the deterioration caused by HIV infection [35–39]. Older heterosexual males are likely less aware about their HIV exposure and IDUs take less care of themselves delaying the initiation of their treatments. Other studies have linked to higher age at HIV diagnosis with worse survival rates [40, 41]. The burden of disease in HIV-infected subjects is thus heavier in older patients [42, 43]. In fact, CD4 cell count at HIV diagnosis is the predominant prognostic factor for survival [33, 44]. Baseline counts of CD4 are more relevant for HIV-related causes of death. This observation has important implications for the clinical management of the patients [44].

#### Survival in Spanish-born and immigrants

The better survival in immigrants, observed in this study, may be due to follow-up losses in those who left their residence in Barcelona [29]. This may be a source of informational bias in our study [45]. It has been found, with respect to other illnesses, that patients with deteriorating condition have the tendency to go back to their countries [46, 47]. We did not find a statistically significant difference in HIV-related causes of death while we did find a difference in non-HIV-related causes. Less deterioration in their condition might be behind the loss of follow-up of these patients. Difference in mortality between immigrants and Spanish-born is also observed in general population. For example, in 2013 0.22% of deaths were registered among immigrants and 0.97% of deaths among Spanish-born, both living in Barcelona [30]. In addition, 16.2% of immigrants were delisted because of leaving Barcelona and Spain [30]. Taking into account that deaths in Spanish category were 8.7% of our study population and assuming the same mortality proportion for immigrants, we might not detect 1.4% of deaths among immigrants group. Although this number would be lower as immigrants were shown to be statistically significantly younger than Spaniards.

#### Educational level

Educational level is an approximation to socio-economic status reflecting social health inequalities. Lower educational level has been related to higher mortality rates from most causes in high-income countries [15, 48]. This gap also occurs among our HIV patients, receiving universal health care [15, 49]. Low educational level may be associated with poor adherence to HAART and worse self-care [50, 51]. Individuals with HIV may have more prevalent risk behaviours [52], for example, this has been observed with smoking [53]. Therefore, low educational level has been related to life-style mortality (non-HIV-related and external causes) but does not derive from HIV-related risk factors, i.e. low educational level is not associated with late presentation of HIV, time to HAART initiation, or HAART response [54]. Despite this we found association among the lowest educational level, the lowest baseline CD4 and to be UDI male with HIV-related causes of death.

#### Survival and district household income

We did not find worse survival according to household income which might be because of the universal access to HAART provided by the Spanish national health system. Other authors have, however, found socioeconomic status, including work status to be an important prognostic risk factor [55–57]. We expected to find worse survival in low-medium household income districts which our Unit had previously observed using other neighbourhood deprivation indicators [29]. Indeed, a national survey in Spain showed that employment among HIV patients was low (45.8%) [58]. Additionally, some of responders in this survey were living in prison (3.0%), other closed facilities (5.0%) or were homeless (1.8%).

#### IDU and heterosexual survival

We found that being IDU was a risk factor for death, which further confirmed the results of previous studies carried out in our unit [28, 29]. IDU male presented higher risk of death by HIV-related causes while IDU female presented higher risk by non-HIV-related causes. Heterosexuals, male and female, also had higher risk of death from non-HIV-related causes than MSM. Our findings may relate to worse HAART adherence [59, 60], or to later HAART initiation [33, 34]. IDUs are diagnosed at an early stage of their infection, but they delay HAART initiation by one or two years. During this time the reduction in their CD4 counts has already reduced their life expectancy [39, 61–64]. Support programmes are therefore necessary to encourage early HAART initiation in this group. On the other hand, heterosexual males have impaired survival due to late diagnosis [29, 39]. For this group, actions aimed at increasing the awareness of and accessibility to HIV testing should be conducted. On the other hand, female risk of death is associated more with non-HIV-related causes which might be because of earlier HAART initiation avoiding HIV-related infections.

### Limitations

The main limitation of our study was that outcome identification required using secondary data sources. The municipal registry and death registry used only captured deaths in the city of Barcelona. We could not identify deaths which occurred outside of the Catalonia region. Despite this, we have found a slightly higher proportion of deaths (5.8%) than a) a Swiss cohort with patients starting HAART during 2005–2009 (5.1%) [27], b) a collaborative cohort composed of HIV-1-infected patients following HAART between 1996 and 2006 from 13 cohorts of Europe and North America (4.8%) [31], and c) Spanish cohorts of the AIDS Research Network from 1997 to 2010 (3.6%) [17]. General population of the municipal registry show a migratory movement surrounding 3% between 2009 and 2013. Assuming a roughly similar behaviour of HIV population without to have into account age, origin and transmission HIV category this might mean the no detection of 8.7% of deaths among a hypothetical 3% of patients leaving Barcelona in our study population resulting in not detecting 6 deaths. Although loss to follow-up would be lower because access to HAART is free in Spain and Barcelona has some of the best high quality hospitals of the country.

We did not find a specific cause of death in 18.7% cases. A sensitivity analysis did not show statistically significant difference between patients with known and unknown specific cause of death when comparing by sex, age, country of origin, household income, HIV transmission category, CD4 counts, viral load and presence of antibodies anti-HCV. Thus, we may not expect changes interpreting Fine and Gray model results if we knew the specific cause for all deaths. Nevertheless, we found difference in educational level. Patients with unavailable cause of death had lower proportion of lower studies completed and higher percentage with unknown educational level. Assuming patients with unknown educational level were patients with low educational level we also might not expect changes interpreting Fine and Gray models.

The evolution of the HIV infection depends on early HAART initiation and good adherence to treatment. Firstly, the criteria for HAART initiation have not always been the same; they change over our study period. For example, the criteria for HAART initiation in 2013 in Spain was having a CD4 count of < 500 cells/μL in asymptomatic patients or presenting an AIDS-defining illness [65]. This means that for our survey and according to CD4 counts, 1680 patients (65.1% of the subjects with CD4 known values) should have started HAART shortly after knowing their HIV diagnosis. However, they did not in fact initiate HAART because the criteria suggested by guidelines in 2002 was to start with CD4 counts < 200 cells/μL and in 2009 was to start with CD4 counts < 350 cells/μL [66, 67]. Further, our database did not include data about HAART regimen and adherence. These variables would have helped us to better understand patient histories.

## Conclusion

There are important differences in survival among transmission groups during study period. The analysis of causes of death in HIV patients has shown that, until 2013, the availability of HAART has not benefited all groups equally, probably because IDUs and heterosexual males start HIV treatment later. It seems that these groups have severe immunodeficiency by the time they approach healthcare services for treatment. Awareness programs and incentives are needed to improve this situation.

Another suggestion, which can be made with respect to the results of this study, is that elderly HIV-patients may require more focused clinical care that extends beyond HIV treatment.
